# Imaging VEGF receptor expression to identify accelerated atherosclerosis

**DOI:** 10.1186/s13550-014-0041-7

**Published:** 2014-08-01

**Authors:** Yared Tekabe, Maria Kollaros, Adam Zerihoun, Geping Zhang, Marina V Backer, Joseph M Backer, Lynne L Johnson

**Affiliations:** Department of Medicine, Columbia University Medical Center, 622 West 168th St, PH 10 center rm 203, New York, 10032 NY USA; SibTech Inc, Brookfield, 06804 CT USA

**Keywords:** Vascular endothelial growth factor, Atherosclerosis, Molecular imaging

## Abstract

**Background:**

The biology of the vulnerable plaque includes increased inflammation and rapid growth of vasa vasorum, processes that are associated with enhanced vascular endothelial growth factor (VEGF)/ imaging receptors for VEGF (VEGFR) signaling and are accelerated in diabetes. This study was designed to test the hypothesis that VEGFRs in atherosclerotic plaques with a SPECT tracer scVEGF-PEG-DOTA/^99m^Tc (scV/Tc) can identify accelerated atherosclerosis in diabetes.

**Methods:**

Male apolipoprotein E null (ApoE^−/−^) mice (6 weeks of age) were made diabetic (*n* = 10) or left as non-diabetic (*n* = 13). At 26 to 28 weeks of age, 5 non-diabetic mice were injected with functionally inactivated scV/Tc (in-scV/Tc) that does not bind to VEGF receptors, while 8 non-diabetic and 10 diabetic mice were injected with scV/Tc. After blood pool clearance, at 3 to 4 h post-injection, mice were injected with CT contrast agent and underwent SPECT/CT imaging. From the scans, regions of interest (ROI) were drawn on serial transverse sections comprising the proximal aorta and the percentage of injected dose (%ID) in ROIs was calculated. At the completion of imaging, mice were euthanized, proximal aorta explanted for gamma well counting to determine the percentage of injected dose per gram (%ID/g) uptake and immunohistochemical characterization.

**Results:**

The uptake of scV/Tc in the proximal aorta, calculated from SPECT/CT co-registered scans as %ID, was significantly higher in the diabetic mice (0.036 ± 0.017%ID) compared to non-diabetic mice (0.017 ± 0.005%ID; *P* < 0.01), as was uptake measured as %ID/g in harvested aorta, 1.81 ± 0.50%ID/g in the diabetic group vs. 0.98 ± 0.25%ID/g in the non-diabetic group (*P* < 0.01). The nonspecific uptake of in-scV/Tc in proximal aorta was significantly lower than the uptake of functionally active scV/Tc. Immunostaining of the atherosclerotic lesions showed higher expression of VEGFR-1 and VEGFR-2 in the diabetic mice.

**Conclusion:**

These initial results suggest that imaging VEGFR with scV/Tc shows promise as a non-invasive approach to identify accelerated atherosclerosis.

**Electronic supplementary material:**

The online version of this article (doi:10.1186/s13550-014-0041-7) contains supplementary material, which is available to authorized users.

## Background

Rupture of an atherosclerotic plaque in the coronary or carotid arteries can lead to sudden and lethal events. A non-invasive imaging approach for detection of atherosclerotic plaques that are prone to rupture would allow for timely preventive treatment and therefore would have a significant clinical impact. In addition to targeting plaque inflammation with ^18^FDG, a number of specific biomarkers, such as P-selectin, VCAM, metalloproteinases, and integrins have been investigated for imaging in mouse models and in human endarterectomy specimens [1]-[7].

Key characteristics of vulnerable plaques include rapid plaque growth accompanied by outward vessel remodeling without increases in luminal narrowing, increased inflammation, and rapid growth of both the hypoxic central core and adventitial vasa vasorum [8]. As expected for pathologies involving inflammation and hypoxia, plaque development is associated with increase in the expression of vascular endothelial growth factor (VEGF) and its receptors (VEGFRs) by endothelial cells, macrophages, and other plaque constituent cells. VEGF/VEGFR signaling has been implicated in the development of atherosclerosis and plaque vulnerability [8]-[14] particularly in association with diabetes [15]-[17].

Since the prevalence of VEGFRs, specifically VEGFR-1 and VEGFR-2, in vulnerable plaques is increased relative to more stable ones, we hypothesized that VEGFR imaging might provide a useful biomarker for assessing plaque vulnerability. To test this hypothesis we used SPECT molecular imaging for quantifying VEGFR prevalence in plaques in apolipoprotein null (ApoE^−/−^) mice in which complex plaque development is accelerated by induction of diabetes.

For VEGFR imaging, we employed previously described scVEGF-PEG-DOTA/^99m^Tc SPECT tracer, (scV/Tc), based on an engineered single-chain (sc) recombinant VEGF, site-specifically derivatized with PEGylated chelator DOTA [18]-[21]. scVEGF-PEG-DOTA conjugate appears to be a versatile platform for the developing of VEGFR-targeting imaging tracers and radiopharmaceuticals. Various scVEGF-based imaging tracers retain nanomolar affinity to VEGFRs, are readily internalized upon binding to the receptors, and reliably detect VEGFR in animal models of cancer [22],[23], aortic aneurism [24], graft atherosclerosis [25], and inflammation [26],[27]. Uptake of scVEGF/Cy5.5 by NIRF imaging of in human carotid endarterectomy specimens localized to expression of VEGFR1 and VEGFR2 and histological characteristics of intraplaque capillary density and infiltrating macrophages [28]. Importantly, the nonspecific uptake of scVEGF-based tracers is evaluated using corresponding tracers based on functionally inactivated scVEGF that does not bind to VEGFRs [19].

## Methods

### Animals

All animal studies were performed with the approval of the Institutional Animal Care and Use Committee of Columbia University. Male C57BL/6 mice and ApoE^−/−^ mice with the genetic background of C57BL/6 mice were purchased from The Jackson Laboratories (Bar Harbor, ME, USA).

### Induction of diabetes

At 6 weeks of age, mice were made diabetic *via* 5 consecutive daily doses of streptozotocin (Sigma, 50 mg/kg in citrate buffer, pH 4.5; St. Louis, MO, USA). Blood glucose levels were monitored weekly *via* tail vein sampling using blood glucose monitor (FreeStyle Lite, Abbott, North Chicago, IL, USA). The mean blood glucose level for the duration for diabetic mice was 309 ± 39 mg/dL. Animals were studied at 26 to 28 weeks of age. On arrival, all of the apoE−/−mice had similar weights, but by the time of study, the diabetic mice were smaller than the non-diabetic mice with average weights of 23.1 ± 1.2 and 30.7 ± 1.1 g (*P* = 0.0001).

### Radiotracer preparation

scV/Tc is based on scVEGF protein which combines two 3 to 112 fragments of human VEGF fused head to tail and is expressed with an N-terminal 15-aa cysteine-containing tag (Cys-tag) for site-specific conjugation [19]. scVEGF-PEG-DOTA and its inactivated analog that lacks VEGFR-binding ability were prepared as described [19]. Briefly, scVEGF was site-specifically derivatized on C4 in Cys-tag with radionuclide chelator 1,4,7,10-tetraazacyclododecane-1,4,7,10-tetraacetic acid (DOTA) *via* 3.4 kDa PEGylated linker. The inactivated VEGF-PEG-DOTA was prepared by biotinylation of 10 to 12 lysine ε amino groups in scVEGF moiety. For ^99m^Tc labeling, lyophilized 0.15 mg scVEGF-PEG-DOTA (approximately 5 nmol) was reconstituted with 75 μl of 0.1 M NaOAc buffer (pH 5.5) and added to 30 to 40 mCi (1,110 to 1,480 MBq) of ^99m^Tc. The mixture was purged with N_2_ for 2 min followed by addition of 50 μg of tin-tricine in 450 μl of deionized water, briefly purged with N_2_ and incubated for 20 min at 55°C. Free ^99m^Tc was separated on PD-10 column (Pharmacia) equilibrated with PBS/0.1% BSA. Fractions (0.5 ml) containing the tracer in the void volume were pooled. The radiochemical purity of scV/Tc was 97% ± 1.6% with the specific activity of 109 ± 11 μCi/μg. The inactivated scVEGF-PEG-DOTA was similarly labeled with ^99m^Tc and the resulting tracer was named in-scV/Tc.

### Blood pool clearance and biodistribution

Blood pool clearance was measured in 3 C57BL/6 mice and 3 ApoE^−/−^diabetic mice. Blood samples were taken by tail vein knicking at 5, 10, 30, 60, 90, 200, 250, and 350 min. Biodistribution of scV/Tc in non-target organs was analyzed in diabetic and non-diabetic mice after imaging. At necropsy, organs were removed, weighed, and counted in the well counter (Wallac Wizard 1470, PerkinElmer, Waltham, MA, USA).

### SPECT/CT imaging

Non-diabetic ApoE^−/−^ mice (*n* = 8) and diabetic ApoE^−/−^ mice (*n* = 10) mice were anesthetized with isofluorane (4% to induce, 1% to maintain) and injected with scV/Tc 0.38 ± 0.08 mCi (14.06 ± 2.92 MBq) *via* femoral vein catheter. To assess nonspecific (non VEGFR-mediated) tracer uptake, a separate group of non-diabetic ApoE^−/−^ mice (*n* = 5) were injected with in-scV/Tc (tracer control). To assess uptake of tracer in non-diseased mice, 2 C57BL/6 mice were injected with scV/Tc (disease control). Three to 4 h after tracer injection (blood pool clearance), all mice were re-anesthetized and injected with 150 to 200 μl (160 mg/mL) of eXIA contrast agent (Binitio Biomedical, Ottawa, Canada) and immediately underwent SPECT/CT imaging on nanoSPECT/CT system (Bioscan, Washington DC, USA).

CT images were acquired with an integrated CT scanner using an X-ray tube at 45 kVp and an exposure time of 1,000 ms per view. The helical SPECT scans were acquired using dual-headed detectors each outfitted with collimators with nine pinholes. Each pinhole had a diameter of 1.4 mm with each collimator providing a transaxial field-of-view (FOV) of 30 mm and an axial FOV of 16 mm, extendable through helical scanning to 270 mm. SPECT data were acquired with the following parameters: step and shoot rotation, 30° step in 360° rotation using 24 projections, 60 s per projection, 256 × 256 frame size with 1.0 mm pixels, and 140 keV with 10% energy window. The obtained projection data were reconstructed by ordered subset expectation-maximization algorithm with subset and iteration number set to 16 and 8, respectively, and a voxel size of 300 μm and SPECT and CT datasets fused. At the end of imaging, mice were euthanized by an intraperitoneal injection of pentobarbital (100 mg/kg).

### Image analyses

**Figure 1:**
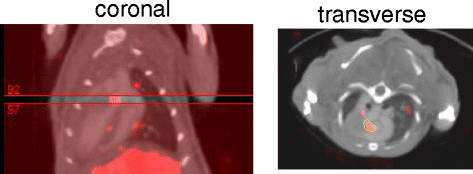
**Method for ROI placement for quantification.** Slices comprising the focal uptake (red color table) are identified on the coronal projection, and an ROI is drawn around the uptake on the transverse projection. The counts in this region are converted to mCi using a calibration standard that is loaded into the software.

The scans were reconstructed and processed using InVivoScope software (Invicro, Boston, MA, USA). Tracer uptake identified as focal areas of activity in the ascending aorta, arch, and brachiocephalic trunk were identified on transverse sections by triangulating from coronal and/or sagittal images and using contrast in the vessels on the CTA for anatomical localization. Regions of interest (ROIs) were drawn around all these areas of focal activity and counts in the ROIs converted to mCi using a calibration algorithm and activity for all regions summed for the total vascular tracer uptake (Figure 1). The summed activity from the ascending aorta and arch (excluding the brachiocephalic vessels) was correlated with *ex vivo* gamma counting of the same tissue. To address whether there were differences in blood pool activity between the diabetic and non-diabetic mice at the time of imaging, ROIs were drawn in the center of the mid-LV cavity on the transverse slice to measure activity (in mCi/mm^3^).

### *Ex vivo* gamma well counting

The chest was opened and the proximal aorta and arch and brachiocephalic vessels photographed *in situ*. Due to time-consuming and technical challenges in dissecting the brachiocephalic vessels, the proximal aorta and arch showing plaque was dissected for correlation with activity measured on scans for the same anatomical segments. The fragment were excised and washed in PBS and weighed, and the radioactivity was counted in a gamma well counter (Wallac Wizard 1470, PerkinElmer, Waltham, MA, USA) and expressed as the percentage of injected dose per gram (%ID/g) of tissue. Radiotracer accumulation in non-target organs was determined similarly.

### Histology and immunohistochemistry

The proximal aorta was harvested and fixed for 24 h in formalin (10%) and paraffin-embedded. Tissue blocks were sectioned (5-μm-thick) and stained with hematoxylin-eosin (H&E) for morphological evaluation. Serial sections (5-μm-thick) were deparaffinized in xylene, treated with 0.3% hydrogen peroxide for 20 min, followed by incubation in protein-free block (Dako, Carpinteria, CA, USA) for 10 min to inhibit the nonspecific binding of the primary antibody. Sections were stained for macrophage marker Mac-3 (RM0029-11H3, rat monoclonal antibody, 1:50 dilution, Santa Cruz Biotechnology, Dallas, TX, USA), VEGFR-1 (rabbit monoclonal antibody, 1:100 dilution, Abcam, Cambridge, MA, USA), VEGFR-2 (rabbit monoclonal antibody, 1:1,500 dilution, Cell Signaling Technology, Beverly, MA, USA), and pan-endothelial cell marker FVIII (rabbit polyclonal antibody, 1:250 dilution, Dako, Carpinteria, CA, USA). Detection was performed with HRP-conjugated respective secondary system followed by diaminobenzidine (DAB substrate kit for peroxidase, Vector Laboratories) and counterstaining with Gill's hematoxylin solution.

Morphometric and immunohistochemical analyses of the arterial segments were performed using a Nikon microscope and Image-Pro Plus software (Media Cybernetics Inc., Silver Spring, MD, USA). The plaque area in the proximal aorta was measured as percent lesion area per total area of the aorta. The positively stained area in the lesion for each marker was calculated as percentage of immunostained area per total area of the vessel.

### Immunofluorescence

To determine the cell types expressing VEGFR-1 and VEGFR-2 in aortic lesions, dual fluorescent confocal microscopy studies were undertaken. Briefly, aortic sections (5-μm-thick) were stained for VEGFR-1 and VEGFR with the respective antibodies as described above. The slides were then incubated with fluorescent tagged secondary antibodies (Texas Red) and co-stained with fluorescent antibodies for endothelial cells (anti-FVIII, 1:200; fluorescein isothiocyanate) or macrophages (anti-Mac-3, 1:50; fluorescein isothiocyanate). The images were examined using confocal fluorescence microscope (Nikon, Melville, NY, USA) and SPOT imaging software (Diagnostic Instruments, Inc., Sterling Heights, MI, USA).

### Statistical analysis

Data are reported as means ± standard deviation. Statistical comparisons between the diabetic and non-diabetic groups and between the inactivated and active tracers were made using Welch's *t* test for groups of unequal size and variance. Estimated power to detect an observed twofold difference between groups of non-diabetic mice imaged with inactivated tracer (*n* = 5) vs. active tracer (*n* = 8) is better than 0.8; and between groups of non-diabetic (*n* = 8) vs. diabetic mice (*n* = 10) imaged with active tracer is better than 0.95. Differences between the groups were considered significant at a value of *P* < 0.05. Correlation for individual values for tracer uptake between scan and well counting was assessed using the Pearson product-moment correlation coefficient.

## Results

### Blood pool clearance and biodistribution

**Figure 2:**
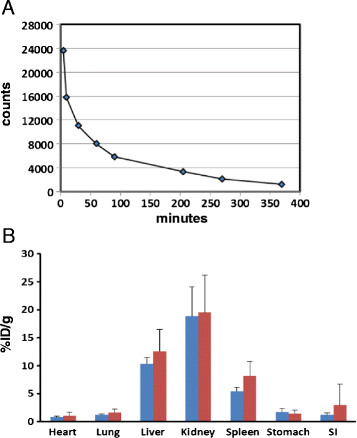
**Blood pool clearance and biodistribution of scV/Tc. A**. Blood pool clearance of scV/Tc in diabetic mice. Each time point represents an average of three mice. **B**. Similar biodistribution of scV/Tc in non-targeted organs in non-diabetic (blue bars) and diabetic (red bars) mice. Bars represent mean ± SD. SI = small intestine.

The clearance of scV/Tc in diabetic ApoE^−/−^ and C57BL/6 mice was fitted to similar biexonential curves with t_1/2_ for the first component of the curve 10 min and for the second 150 min (Figure 2A for diabetic ApoE^−/−^ mice). The biodistribution of scV/Tc in non-target organs of non-diabetic and diabetic ApoE^−/−^ mice was found to be similar, with highest uptake in the kidneys and liver (Figure 2B).

### Scans

**Figure 3:**
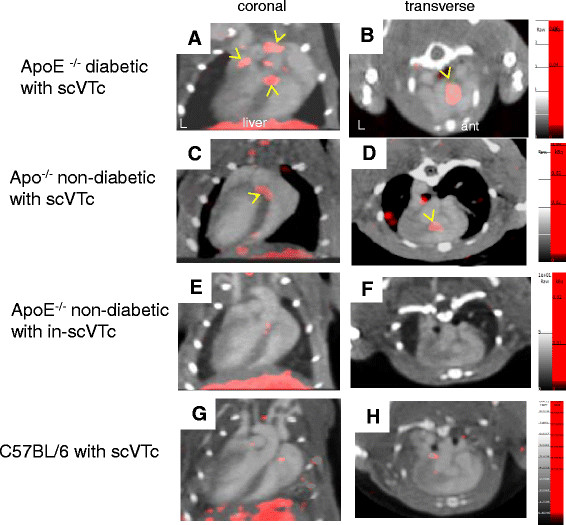
**SPECT/CT imaging of atherosclerotic plaques in diabetic and non-diabetic ApoE**^**−/−**^**mice.** Representative coronal (left) and transverse (right) images obtained for diabetic ApoE^−/−^ mouse **(A, B)**, non-diabetic ApoE^−/−^ mouse **(C, D)** injected with functionally active scV/Tc, for non-diabetic ApoE^−/−^ injected with functionally inactivated in-scV/Tc **(E, F)** for assessment of nonspecific, not VEGFR-mediated tracer uptake, and C57BL/6 mouse **(G, H)** injected with scV/Tc for assessment of tracer uptake in non-atherosclerotic, non-diabetic mouse (disease control). The CT contrast outlines the ventricular cavities and arterial vessels. Focal areas of scV/Tc uptake (in red) are found in the aortic arch and proximal brachiocephalic branches in diabetic mice, but are localized mostly to the aortic root in the non-diabetic mouse, and yellow arrows indicate the prominent areas of tracer uptake. No appreciable uptake of tracer localized to vascular territories was seen in the ApoE^−/−^ mice injected with in-scV/Tc or the C57BL/6 mice injected with scV/Tc.

Scans from diabetic and non-diabetic ApoE^−/−^ mice showed unequivocal uptake of scV/Tc in the proximal aorta and arch on the co-registered CT scan (Figure 3A,B,C,D as representative scans) with tracer uptake extending into the brachiocephalic vessels in the diabetic mice (compare representative scan Figure 3A,B for diabetic vs. Figure 3C,D for non-diabetic). ApoE^−/−^ mice injected with in-scV/Tc and C57BL/6 mice injected with scV/Tc showed little or no tracer uptake in areas corresponding to the location of the proximal aorta and arch on the co-registered SPECT/CT scan (Figure 3E,F,G,H).

The blood pool activity levels determined from the scans for the non-diabetic and diabetic mice were not significantly different: 18.6 × 10^–3^ vs. 19.4 × 10^−3^ mCi/mm^3^ (*P* = 0.86).

### Lesion size

The mean cross-sectional area of the proximal aortic lesions, expressed as percent lesion area over total aortic area, in the diabetic group (32.3% ± 6.05%) was significantly larger than in the non-diabetic group receiving scV/Tc (15.3 ± 4.3%; *P* < 0.01) The lesion size in the non-diabetic ApoE^−/−^ mice receiving the in-scV/Tc (12.0% ± 2.9%) was not statistically different from the non-diabetic mice receiving scV/Tc.

### Tracer uptake quantitation

**Figure 4:**
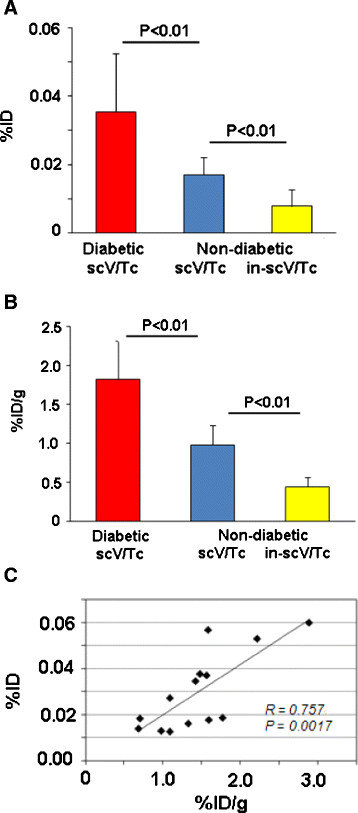
**Quantitative analysis of tracer uptake. (A)** Average %ID ± standard deviation for uptake of functionally active scV/Tc in diabetic (red bar), non-diabetic (blue bar), and nonspecific (non-VEGFR-mediated) in-scV/Tc uptake in non-diabetic ApoE^−/−^ mice (yellow bar). **(B)** Average %ID/g for the same groups of mice, as calculated from gamma well counting of harvested aorta. **(C)** Correlation for %ID vs. %ID/g.

The average uptake of scV/Tc or in-scV/Tc in thorax areas corresponding to the proximal aorta, arch, and brachiocepahalic vessels was calculated from scans as %ID, as described in Methods (Figure 4A). The average uptake of scV/Tc in diabetic ApoE^−/−^ mice was significantly higher than in non-diabetic ApoE^−/−^ mice (0.036 ± 0.017 vs. 0.017 ± 0.005%ID; *P* < 0.01) (Figure 4A). The non VEGFR-mediated uptake of in-scV/Tc in ApoE^−/−^ mice, was significantly lower than that of functionally active scV/Tc in age-matched ApoE^−/−^ mice with similar size lesions (0.008 ± 0.005 vs. 0.017 ± 0.005%ID; *P* < 0.01).

These findings were confirmed by ex-vivo gamma well counting of harvested fragments of aorta with the most conspicuous plaques (Figure 4B). Uptake of scV/Tc calculated for these fragments as %ID/g in diabetic ApoE^−/−^ mice (1.81 ± 0.50%ID/g) was significantly higher than that in the non-diabetic ApoE^−/−^ group (0.9 ± 0.25%ID/g, *P* < 0.01). In fragments harvested from non-diabetic ApoE^−/−^ mice with similar extent of atherosclerosis the nonspecific (non VEGFR-mediated) uptake of in-scV/Tc (0.44 ± 0.12%ID/g) was significantly lower than scV/Tc uptake (P < 0.01). Tracer uptake in the proximal aorta and arch of each mouse, determined as %ID from scans, plotted vs. %ID/g (well counter) determined for the same anatomical aortic section in the same mouse correlated significantly, (*r* = 0.75, *P* = 0.0017) (Figure 4C).

### Histology and quantitative immunohistolchemistry

**Figure 5:**
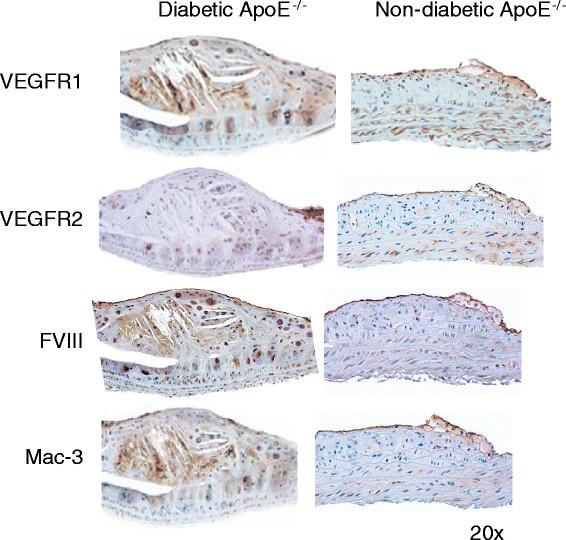
**Immunohistochemical characterization of atherosclerotic plaques.** Representative aortic tissue sections stained for VEGFR-1, VEGFR-2, FVIII (marker for endothelial cells), and Mac-3 (marker for macrophages) in diabetic and non-diabetic ApoE^−/−^ mice.

**Figure 6:**
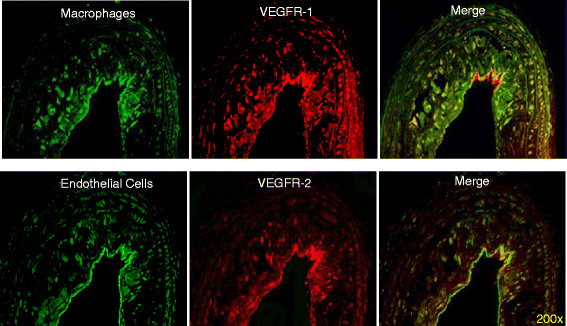
**Differential expression of VEGFR-1 and VEGFR-2 on endothelial cells and macrophages.** Double immunofluorescent staining for VEGFRs and markers of endothelial cells (FVIII) and macrophages (Mac-3). Serial atherosclerotic sections from diabetic mice were double-stained for VEGFR-1 and Mac-3 (upper row) or VEGFR-2 and FVIII (lower row), as indicated. Co-localization is visible on merged images as the yellow color.

Lesions from the diabetic ApoE^−/−^ mice showed complex plaque anatomy with cholesterol clefts whereas lesions from age-matched non-diabetic ApoE^−/−^ mice were at an earlier (fatty streak) stage. Immunostaining of serial sections through the plaque in the proximal aorta revealed greater staining for VEGFR-1, VEGFR-2 in the diabetic group than in the non-diabetic group, and this staining was observed on the luminal aorta surface as well as in the inner plaque area enriched in cells stained for endothelial marker FVIII and macrophage marker Mac-3 (Figure 5). Quantitative immunohistochemistry of the aortic tissue revealed greater expression of VEGFR-1 in the diabetic mice (9.17% ± 4.46%) compared with the non-diabetic (1.88% ± 1.48%; *P* < 0.01). Similarly, VEGFR-2 expression was significantly higher in the diabetic mice (3.25% ± 1.27%) compared with the non-diabetic (1.31% ± 0.86%; *P* < 0.01). Total macrophage burden in the atherosclerotic lesions of diabetic mice (5.85% ± 2.86%) was also significantly higher compared with the non-diabetic mice (0.99% ± 0.38%; *P* < 0.01). Immunofluorescence studies showed co-localization of VEGFR-1 primarily with macrophage marker Mac3 and VEGFR-2 with endothelial cell marker FVIII (Figure 6).

## Discussion

This is the first study to report the results of *in vivo* SPECT imaging of a ^99m^Tc-labeled VEGFR-targeting molecular tracer (scV/Tc) to detect more advanced complex lesions in diabetic ApoE^−/−^mice compared to age matched non-diabetic ApoE^−/−^ mice with fatty streaks. We showed that the scV/Tc uptake is predominantly VEGFR-mediated by showing significantly lower uptake of in-scV/Tc, incapable of VEGFR binding.

According to the current models of plaque progression, enhanced VEGF/VEGFR signaling plays an important role in three critical processes leading to plaque vulnerability: stimulation of angiogenesis in plaque, recruitment of monocytes into plaque, and increasing permeability of plaque vasculature which leads to hemorrhage and inflammatory cell extravasation [8],[10]. Importantly, studies of accelerated atherosclerosis and enhanced plaque vulnerability in human diabetic patients, as well as in diabetic rabbit and mouse atherosclerosis models, revealed higher prevalence of VEGF and VEGFRs in diabetic vs. non-diabetic plaques [15]-[17]. Immunohistochemical analysis confirmed that the significantly higher uptake of the functionally active scV/Tc in the more advanced and complex atherosclerotic lesions in diabetic vs. non-diabetic ApoE^−/−^ mice was due to higher prevalence of both VEGFR-1 and VEGFR-2. Although in this study, we did not image ApoE^−/−^ mice fed with high-fat (Western) diet to accelerate atherosclerosis since the lesion histology in the diabetic mice is very similar to that of mice at the same age fed with high-fat (Western) diet [29].

Some variation in nonspecific uptake of the inactivated in-scV/Tc tracer was noted among mice; however, the level of uptake was significantly lower than receptor-mediated uptake of scV/Tc. This small amount of nonspecific trapping of a protein-based tracer was probably a result of the enhanced permeability and retention effects in inflammatory atherosclerotic tissue.

The diversity of VEGFR-expressing cells raises the question which cells are responsible for scV/Tc uptake. In agreement with other studies [10],[14],[28], our immunohistochemical analysis indicates that in atherosclerotic plaques, VEGFR-2 receptors in this ApoE^−/−^ model are predominantly expressed on easily accessible luminal endothelial cells, while VEGFR-1 receptors are predominantly expressed on intraplaque macrophages. In human atherosclerosis, endothelial cells are also found in neoangiogenesis in the adventitia (vasa vasorum) and media in coronary and carotid atherosclerotic plaques associated with plaque vulnerability. These leaky capillaries express VEGR-2 on endothelial cells. The combined binding of scV/Tc to both macrophages and endothelial cells in plaque neoangiogenesis in theory provide two targets for scV/Tc binding to boost plaque signal [8],[10],[30]. Image analysis of VEGFR-1 and VEGFR-2 independently in atherosclerotic plaques using tracers based on the known VEGF mutants [31] with predominant affinity to one receptor might further hone our ability to specifically target neoangiogenesis.

Judging by the findings in mouse aortic aneurism model [24], mouse graft arteriosclerosis model [25], and mouse urine bladder inflammation model [26], a relatively small (approximately 29 kDa) fluorescent scVEGF tracer can be taken up not only by readily accessible luminal endothelial cells but also by other VEGFR-expressing cells. We expect that a similarly small (32 kDa) scV/Tc tracer is also able to cross the vascular barrier and distribute between various VEGFR-expressing cells. In this respect, imaging with scV/Tc potentially provides more target cells than ultrasound agents [23],[28] or MRI agents (unpublished data) that, due to their relatively large size, are confined to the intravascular space targeting only VEGFRs on luminal endothelial cells. Of note, quantitation of scV/Tc uptake, either from scans or from gamma counting of tissue samples, provides only the ‘average’ uptake values, and do not reflect significant heterogeneity in VEGFR expression, as detected by immunohistochemical analysis of our samples (Figures 5 and 6). Similar heterogeneity was observed in harvested human plaques with a fluorescent scVEGF-based tracer [28].

The uptake of ^18^ F-FDG in arterial vessel walls signaling the high metabolic activity of inflammatory cells in atherosclerotic plaque or vasculitis has been observed and referenced extensively in the literature in both animal models and in patients [32]. The binding of scV/Tc to VEGFR-1 expressed on macrophages indicates the similar inflammation binding target as ^18^FDG. Differences include the additional target of neoangiogenesis for scV/Tc and the low constitutive expression of VEGFRs in normal myocardium. Whether these differences will translate into better specificity and signal to noise for scV/Tc vs. ^18^FDG is yet to be determined.

The significance of our findings and, in general, the interest in imaging VEGFRs in atherosclerotic plaques are due to growing appreciation of the role of VEGF/VEGFR signaling and enhanced VEGFR expression in atherosclerosis [10] and in vulnerable plaque [8]. Our VEGFR imaging and immunohistochemical results comparing diabetic and non-diabetic ApoE^−/−^ mice support the emerging role of VEGFR as a biomarker for assessing complex and inflamed atherosclerotic plaques.

## Conclusion

We report the first *in vivo* imaging study of VEGFR prevalence in atherosclerotic plaques in diabetic vs. non-diabetic atherosclerosis-prone ApoE^−/−^ mice, a model where atherosclerosis is accelerated by diabetes. Using scVEGF-PEG-DOTA/^99m^Tc SPECT tracer (scV/Tc) that binds to and is internalized by VEGFR-1 and VEGFR-2, we found enhanced uptake of the tracer in atherosclerotic plaques of diabetic vs. non-diabetic mice. The imaging findings indicate higher prevalence of VEGFRs in accelerated atherosclerosis and this conclusion is supported by immunohistochemical analyses of VEGFRs in corresponding plaques. Because higher prevalence of VEGFRs is associated with inflammation, angiogenesis, and enhanced plaque vulnerability, our findings suggest that VEGFR imaging with scV/Tc may provide a clinically relevant biomarker of plaque vulnerability.

## Authors’ original submitted files for images

Below are the links to the authors’ original submitted files for images. Authors’ original file for figure 1 Authors’ original file for figure 2 Authors’ original file for figure 3 Authors’ original file for figure 4 Authors’ original file for figure 5 Authors’ original file for figure 6
